# Low levels of endogenous or X-ray-induced DNA double-strand breaks activate apoptosis in adult neural stem cells

**DOI:** 10.1242/jcs.171223

**Published:** 2015-10-01

**Authors:** Lara Barazzuol, Nicole Rickett, Limei Ju, Penny A. Jeggo

**Affiliations:** Genome Damage and Stability Centre, University of Sussex, East Sussex BN19RQ, UK

**Keywords:** Neural stem cell, DNA double-strand break repair, Apoptosis, Radiation sensitivity

## Abstract

The embryonic neural stem cell compartment is characterised by rapid proliferation from embryonic day (E)11 to E16.5, high endogenous DNA double-strand break (DSB) formation and sensitive activation of apoptosis. Here, we ask whether DSBs arise in the adult neural stem cell compartments, the sub-ventricular zone (SVZ) of the lateral ventricles and the sub-granular zone (SGZ) of the hippocampal dentate gyrus, and whether they activate apoptosis. We used mice with a hypomorphic mutation in DNA ligase IV (*Lig4^Y288C^*), ataxia telangiectasia mutated (*Atm^−^*^/*−*^) and double mutant *Atm^−^*^/*−*^/*Lig4^Y288C^* mice. We demonstrate that, although DSBs do not arise at a high frequency in adult neural stem cells, the low numbers of DSBs that persist endogenously in *Lig*4^*Y288C*^ mice or that are induced by low radiation doses can activate apoptosis. A temporal analysis shows that DSB levels in *Lig4^Y288C^* mice diminish gradually from the embryo to a steady state level in adult mice. The neonatal SVZ compartment of *Lig4^Y288C^* mice harbours diminished DSBs compared to its differentiated counterpart, suggesting a process selecting against unfit stem cells. Finally, we reveal high endogenous apoptosis in the developing SVZ of wild-type newborn mice.

## Abstract (precis)

**Summary:** This study shows that endogenous and radiation-induced DNA damage sensitively activates apoptosis in the adult sub-ventricular zone (SVZ). Developmentally regulated apoptosis is shown in the neonatal SVZ.

## INTRODUCTION

DNA double-strand breaks (DSBs) are severe lesions that can cause cell death and/or chromosomal rearrangements. They are the major lethal lesion induced by ionising radiation but can also arise from oxidative damage, following replication and during immune development. DNA non-homologous end-joining (NHEJ) is the main DSB repair pathway, with DNA ligase IV (LIG4) being the unique NHEJ ligase. DNA ligase IV knockout in mice is embryonically lethal owing to extensive neuronal apoptosis around embryonic day (E)13.5 (Barnes et al., 1998; Frank et al., 1998). In humans, hypomorphic mutations in DNA ligase IV confer LIG4 syndrome, which is characterised by immunodeficiency, microcephaly and developmental delay (O'Driscoll et al., 2001). A recent study has proposed that DNA ligase IV deficiency is a common cause of extreme growth failure and microcephaly (Murray et al., 2014).

**Fig. 1.**
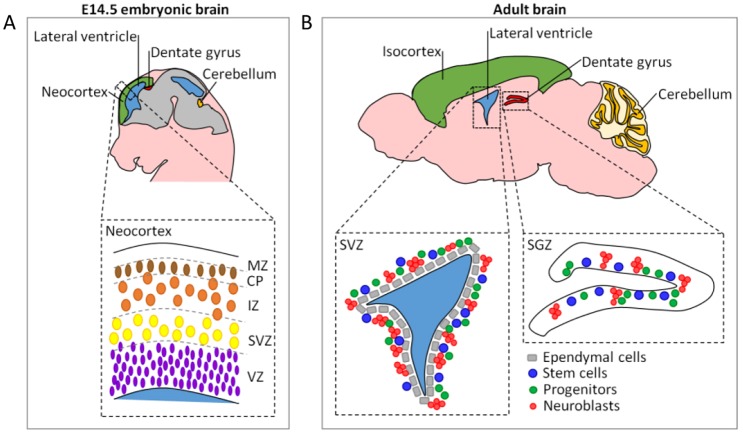
**Schematic representation of the embryonic and adult brain.** (A) Sagittal view of an embryonic E14.5 mouse brain. The dashed line inset represents the neocortex (green) and its location. At this developmental stage, the neocortex can be divided into distinct layers: the ventricular and sub-ventricular zones (VZ/SVZ), which lie adjacent to the ventricles (blue), contain the neural stem and intermediate progenitor cells; the intermediate zone (IZ), which lies above the VZ/SVZ, contains migrating cells and axons; and the cortical plate (CP), between the IZ and the superficial marginal zone (MZ), which is formed of post-mitotic neurons. (B) Sagittal view of an adult mouse brain. During development, the CP progressively expands and gives rise to the adult isocortex (green), leaving behind the adult stem cell SVZ close to the lateral ventricles (left dashed line inset). The sub-granular zone (SGZ) of the dentate gyrus within the hippocampus constitutes another adult stem cell compartment (right dashed line inset). Both of these regions are comprised of three main cell types: in turn, the stem cells, the progenitor cells and the neuroblasts. Note that the equivalent anatomical regions in the upper right panel are illustrated using the same colours as in the upper left panel.

Pioneering studies describing the structure of the embryonic neocortex have shown that the ventricular zone (VZ) and sub-ventricular zone (SVZ), hereafter VZ/SVZ, adjacent to the ventricles, contain the neural stem and intermediate progenitor cells, which replicate rapidly from E11 to E16.5 (Bayer et al., 1991). The intermediate zone (IZ), which lies above the VZ/SVZ, contains migrating cells and axons (Mitsuhashi and Takahashi, 2009; Pontious et al., 2008). At ∼E14, post-mitotic neurons establish a layer known as the cortical plate (CP) between the IZ and the superficial marginal zone (Fig. 1A). Following ionising radiation at doses below 50 mGy, these embryonic neural stem and early progenitor cell compartments activate apoptosis, which we define hereafter as sensitive activation of apoptosis. Studies using DNA-ligase IV-null mice and a strain with a hypomorphic mutation in DNA ligase IV (*Lig4^Y288C^*), which arose from a random mutagenesis screen and causes a diminished rate of DSB repair, have revealed that there are elevated levels of endogenous DSBs and apoptosis in the neocortical VZ/SVZ and IZ (Gatz et al., 2011; Lee et al., 2001; Nijnik et al., 2007). Both the high level of DSBs observed in *Lig4^Y288C^* embryos and number of apoptotic cells diminish temporally following the cessation of rapid proliferation in the VZ/SVZ, suggesting a causal relationship. Collectively, these findings suggest that DSBs arise during neurogenesis and sensitively activate apoptosis in the neocortex. Ionising-radiation-induced apoptosis in the embryonic neocortex is largely dependent upon the damage response kinase ataxia telangiectasia mutated (ATM) (Gatz et al., 2011; Lee et al., 2001; Sekiguchi et al., 2001). In the adult brain, neurogenesis persists in two main regions – the SVZ, adjacent to the lateral ventricle, and the sub-granular zone (SGZ), located in the hippocampal dentate gyrus (Fig. 1B) (Lledo et al., 2006). The sensitivity of the response of the SVZ and SGZ to DNA damage has not been investigated.

Here, we examine whether the adult SVZ and SGZ incur endogenous DSBs and whether low levels of DSBs can activate apoptosis. We examined these endpoints in *Lig4^Y288C^*, *Atm^−^*^/*−*^ and double mutant *Atm^−^*^/*−*^/*Lig4^Y288C^* mice. We observed similar DSB levels in the adult SVZ and SGZ of *Lig4^Y288C^* mice, and this level was also similar to that found in differentiated neuronal compartments, suggesting that, unlike the situation in embryos, DSBs do not arise at high frequency in the adult neural stem cells. However, apoptosis was sensitively activated by DSBs in the SVZ in a predominantly ATM-dependent manner. Thus, sensitive activation of apoptosis in neural stem cells is not a direct consequence of rapid replication but a feature of the compartment. These findings are important when considering the use of radiological procedures. To gain further insight into the generation of DSBs during development and the fate of cells with DSBs generated during embryogenesis, we undertook a temporal analysis in mice which revealed that the level of DSBs gradually decreased from late embryogenesis to shortly after birth, reaching a steady state level by 2 months. Such a temporal loss of DNA damage suggests that cells with DSBs generated during embryonic neurogenesis can progress into the neonatal mouse brain and undergo slow DSB repair. Additionally, the temporal analysis revealed a defined postnatal stage of developmentally regulated and ATM-independent apoptosis that occurs during establishment of the adult SVZ. We provide evidence for reduced DSB levels in the stem cell compartment shortly after birth in *Lig4^Y288C^* mice, suggesting that there is selective loss of unfit stem cells.

## RESULTS

### Increased DSBs in neural stem and differentiated cells of adult *Lig4^Y288C^* mice

**Fig. 2.**
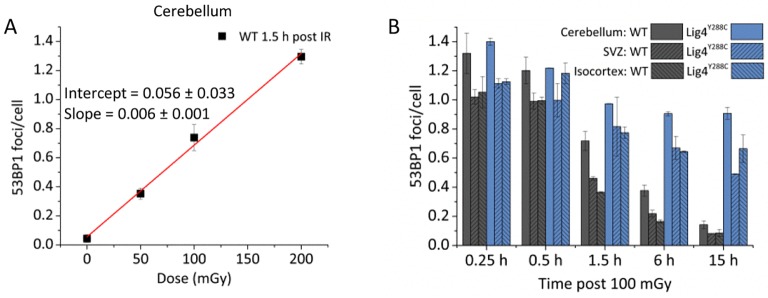
**Assessment of DSB formation and repair.** (A) Quantification of 53BP1 foci at 1.5 h in adult WT cerebellum following exposure to 50–200 mGy X-rays. We examined 53BP1 rather than γH2AX, the more commonly used DSB marker, because γH2AX can form in replicating cells without DSB formation. Data were fitted with a linear no-threshold model. The ANOVA *P*-value for the fit was <0.002. (B) 53BP1 repair analysis in adult WT and *Lig4^Y288C^* cerebellum, SVZ and isocortex following irradiation with 100 mGy X-rays. Data represent mean±s.e.m. (*n*≥2 for each genotype and treatment). The WT and *Lig4^Y288C^* cerebella have slightly enhanced DSB numbers at all time points possibly reflecting enhanced detection or a tissue weighting effect. Note that we observed similar numbers of foci at 15 and 30 min after ionising radiation, as observed using cultured cells, which most likely reflects the fact that foci are both undergoing repair and increasing in size (and hence detection ability) during this time period.

**Fig. 3.**
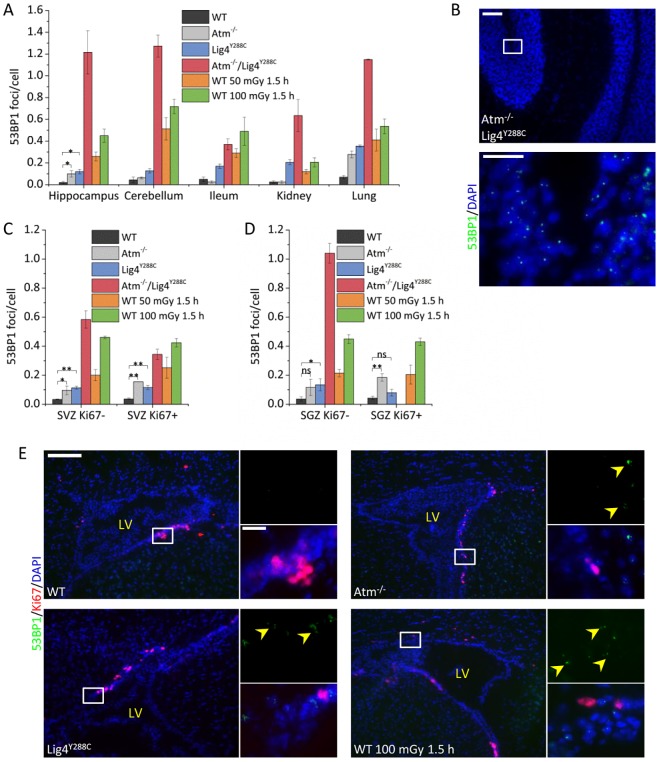
**Similar levels of endogenous DSB formation in differentiated neuronal tissues and adult stem cell compartments.** (A) Quantification of 53BP1 foci per cell in different tissues of untreated adult mice of the genotypes indicated and WT mice exposed to 50 or 100 mGy X-rays. (B) Representative images of untreated *Atm^−^*^/*−*^/*Lig4^Y288C^* cerebellum stained for 53BP1 (green) and DAPI (blue). The lower image represents the boxed area shown in the upper image. Scale bars: 100 µm (top), 20 µm (bottom). (C,D) 53BP1 foci per cell in the adult SVZ and SGZ regions comparing proliferating (Ki67^+^) and non-proliferating (Ki67^−^) cells. Note that 53BP1 foci were not scored in the *Atm^−^*^/*−*^/*Lig4^Y288C^* SGZ due to the low number of Ki67^+^ cells. (E) Representative images of the adult SVZ of untreated WT, *Atm*^−/−^ and *Lig4^Y288C^* mice, and WT SVZ 1.5 h after 100 mGy X-rays stained for Ki67 (red), 53BP1 (green) and DAPI (blue). Scale bars: 150 µm (left), 20 µm (right). The position of the lateral ventricle (LV) is shown for orientation. Yellow arrowheads show 53BP1 foci. Data represent mean±s.e.m. (WT, *n*=4; *Atm^−^*^/*−*^, *n*=3; *Lig4^Y288C^*, *n*=5; *Atm^−^*^/*−*^/*Lig4^Y288C^*, *n*=3; WT 50 mGy 1.5 h, *n*=2; WT 100 mGy 1.5 h, *n*=3). ns, not significant; *P≤0.05; **P≤0.01 (unpaired Student's *t*-test).

Our previous analysis of *Lig4^Y288C^* embryos, which repair DSBs with slow kinetics, has revealed that there is a high level of DSBs in the embryonic neocortex compared to other embryonic tissues (Gatz et al., 2011). First, we examined whether high levels of DSBs are also observed in the adult stem and early progenitor regions by quantifying 53BP1 foci, a DSB marker, in the SVZ and SGZ of wild-type (WT) and *Lig4^Y288C^* mice. To verify the system, we demonstrated that there was a dose-dependent induction of 53BP1 foci in the cerebellum of WT mice and impeded DSB repair in *Lig4^Y288C^* mice (Fig. 2A,B). We then quantified 53BP1 foci in various tissues from adult mice (2–3 months old). Given that we aimed subsequently to examine apoptosis, which is activated by ATM at DSBs, we examined 53BP1 foci in WT, *Lig4^Y288C^*, *Atm^−^*^/*−*^ and double mutant *Atm^−^*^/*−*^/*Lig4^Y288C^* mice. We observed a low level of endogenous 53BP1 foci in WT mice in all tissues examined, and a small, but significant, increase in the level of foci in *Lig4^Y288C^* mice (Fig. 3A, compare black and blue columns). The cerebellum and hippocampus, which are non-replicating, had similar DSB levels to that in the proliferating ileum. Thus, the steady state level of DSBs did not correlate with the proliferative status. In most tissues (except the kidney), 53BP1 foci numbers in *Lig4^Y288C^* mice were approximately half that obtained at 1.5 h after exposure of WT mice to 50 mGy X-rays, suggesting a level of DSBs similar to that induced by 15–20 mGy X-rays (Fig. 3A, compare blue and orange columns). *Atm^−^*^/−^ mice harboured elevated numbers of 53BP1 foci compared to WT mice in the hippocampus and lung but not in the cerebellum, ileum or kidney (Fig. 3A, compare black and light grey columns). 53BP1 foci numbers were similar in the *Atm^−^*^/*−*^ and *Lig4^Y288C^* hippocampus. Strikingly, *Atm^−^*^/*−*^/*Lig4^Y288C^* mice had high numbers of 53BP1 foci in all tissues except the ileum (Fig. 3A,B).

Next, we quantified 53BP1 foci in the adult SVZ and SGZ. In rodents, the SVZ has more extensive germinal activity compared to the SGZ (Alvarez-Buylla et al., 2001; Sanai et al., 2004). The SVZ and SGZ of WT and *Lig4^Y288C^* mice had similar 53BP1 foci numbers to that enumerated in the differentiated cerebellum and hippocampus (Fig. 3A). In addition, the distribution of DSBs was similar in the SVZ, SGZ and cerebellum arguing against there being a subset of replicating cells with high breakage (data not shown). The expression of the cellular marker Ki67 (also known as MKI67) correlates with cell proliferation. 53BP1 foci levels were similar in Ki67^+^ versus Ki67^−^ SVZ and SGZ cells (Fig. 3C,D). DSB detection or formation was slightly reduced in the SVZ compared to the cerebellum, but the rate of DSB repair was similarly impaired in the *Lig4^Y288C^* cerebellum versus the SVZ (Fig. 2B; supplementary material Fig. S1A,B). 53BP1 foci numbers in *Atm^−^*^/*−*^ mice were similar in the SVZ and SGZ compared to the hippocampus, and similar to the level in *Lig4^Y288C^* mice (Fig. 3C,D, light grey columns). Notably, *Atm^−^*^/*−*^/*Lig4^Y288C^* mice had significantly lower numbers of 53BP1 foci in the SVZ compared to the hippocampus and cerebellum, but the numbers were elevated compared to *Lig4^Y288C^* mice (Fig. 3A,C).

Comparison of DSB levels in the adult SVZ and cerebellum of *Lig4^Y288C^* mice with those in the embryonic neocortex has revealed that the levels are approximately ninefold lower in adult mice (Gatz et al., 2011). Indeed, in the embryonic neocortex, the level is similar to that induced by 120 mGy X-rays compared to 15–20 mGy in the adult mice (Gatz et al., 2011). The level of DSBs in *Atm^−^*^/*−*^/*Lig4^Y288C^* adult mice was substantially greater than in *Lig4^Y288C^* single mutant adult mice and similar to the *Lig4^Y288C^* embryos (Fig. 3A,C).

Collectively, these findings demonstrate that elevated DSBs are present in multiple neuronal tissues of *Lig4^Y288C^* mice. However, the level of breakage in these neuronal tissues, including the adult SVZ and SGZ, is lower than in the embryonic neocortex (comparison with Gatz et al., 2011). This suggests that, unlike the embryonic neocortex, the adult SVZ and SGZ are not characterised by high DSB formation, which could reflect their lower level of replication compared to the embryonic counterpart.

### Temporal analysis reveals a gradual decrease in DSB levels in neuronal tissues from embryogenesis through to adulthood

This and previous analyses suggest that at E14.5, the *Lig4^Y288C^* neocortex has approximately one 53BP1 focus per cell, which is estimated to be equivalent to three DSBs per cell (Gatz et al., 2011). To gain insight into the fate of these cells and the origin of DSBs in adult neuronal tissues, we undertook a temporal analysis of DSB levels in neuronal tissues from E14.5 to adulthood (taken as 2–3 months post birth). In the embryonic neocortex at E14.5 and E17.5, we scored 53BP1 foci in the IZ and CP, the early progenitors of the VZ/SVZ, where foci numbers can be most accurately quantified (Gatz et al., 2011). After birth, we examined the isocortex and SVZ, tissues that originate from the embryonic VZ/SVZ (Alvarez-Buylla et al., 2001; Tramontin et al., 2003). Given that perinatally the majority of cells in the adult SVZ are Ki67^+^, we did not distinguish the Ki67 proliferative status in this analysis. Where possible, we examined all genotypes, but owing to the low numbers of living *Atm^−^*^/*−*^/*Lig4^Y288C^* mice born, this analysis was restricted.

**Fig. 4.**
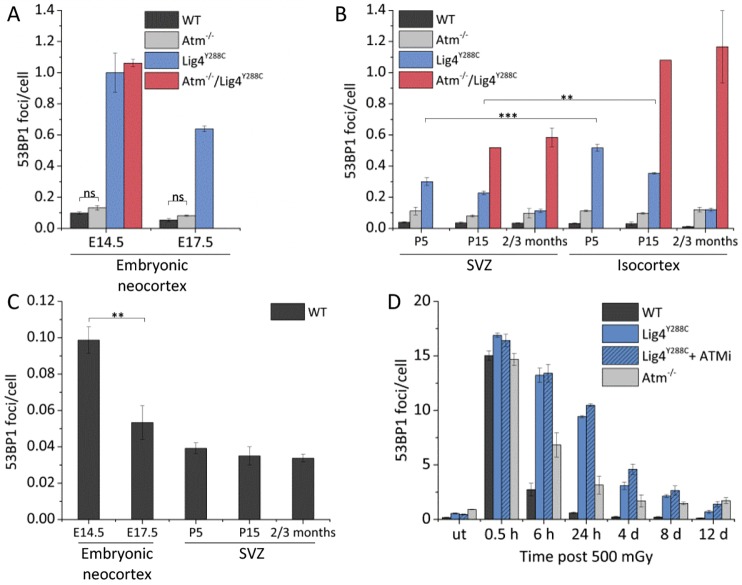
**DSBs generated during embryonic neurogenesis can progress into the isocortex and SVZ of newborn mice and undergo slow DSB repair.** (A,B) 53BP1 foci per cell at different embryonic neurodevelopmental stages in the neocortex (E14.5 and E17.5), postnatal (P5 and P15) and mature (2–3 months) isocortex and SVZ. *Atm^−^*^/*−*^/*Lig4^Y288C^* marked P15 represents P20. All *P* values between WT versus *Atm^−^*^/*−*^ and WT versus *Lig4^Y288C^* at each developmental stage were statistically significant (*P*≤0.05), unless reported otherwise. (C) Larger scale version of 53BP1 foci per cell level in the neocortex and SVZ of WT mice (as shown in A and B). (D) Repair kinetics of MEFs after exposure to 500 mGy in the presence or absence of 10 µM ATMi KU55933. ut, untreated. Data represent mean±s.e.m. (at E14.5 WT, *n*=4; *Atm^−^*^/*−*^, *n*=7; *Lig4^Y288C^*, *n*=3; *Atm^−^*^/*−*^/*Lig4^Y288C^*, *n*=2; at E17.5 WT, *n*=3; Atm^−/−^, *n*=3; *Lig4^Y288C^*, *n*=2; *Atm^−^*^/*−*^/*Lig4^Y288C^*, n/a; at P5 WT, *n*=7; *Atm^−^*^/*−*^, *n*=3; *Lig4^Y288C^*, *n*=4; *Atm^−^*^/*−*^/*Lig4^Y288C^*, n/a; at P15 WT, *n*=2; *Atm^−^*^/*−*^, *n*=3; *Lig4^Y288C^*, *n*=2; at P20 *Atm^−^*^/*−*^/*Lig4^Y288C^*, *n*=1). ns, not significant; *P≤0.05; **P≤0.01; ***P≤0.001 (unpaired Student's *t*-test).

For WT embryos, we observed an approximately twofold greater number of DSBs in the embryonic neocortex at E14.5 compared to E17.5, which we attribute to the fact that replication has substantially diminished by E17.5 and that DSBs arise as a consequence of rapid replication (Fig. 4A,C, black columns). Consistent with previous findings, the level of DSBs in *Lig4^Y288C^* embryos was similar to that induced by 120 mGy X-rays (Fig. 4A, blue columns) (Gatz et al., 2011). In *Lig4^Y288C^* embryos, DSB levels at E17.5 were elevated compared to control embryos, although they were significantly lower than at E14.5 (*P*≤0.05) (Fig. 4A). We attribute this to a diminished rate of DSB formation, given that replication has ceased by E17.5, coupled with slow residual repair in *Lig4^Y288C^* embryos. Despite efficient repair in WT cells, a similar trend is observed, although DSB levels are lower than in *Lig4^Y288C^* embryos (Fig. 4C). *Atm^−^*^/*−*^ embryos displayed a small increase in DSBs at E14.5, the level of which remained similar at E17.5, but the difference was not significant (Fig. 4A, compare black and light grey columns). *Atm^−^*^/*−*^/*Lig4^Y288C^* embryos had a similar increased level of DSBs to *Lig4^Y288C^* embryos at E14.5 suggesting that additional loss of ATM does not enhance DSB formation (E17.5 embryos were not available for analysis) (Fig. 4A, red columns). Taken together, these findings substantiate our previous observations that rapid proliferation from E11 to E16.5 results in high DSB formation, which diminishes when replication declines (i.e. by E17.5) (Gatz et al., 2011).

Examination of DSBs in the WT isocortex of newborn mice [postnatal day (P)5] revealed lower DSB levels compared to those in the embryo, and levels further decreased by 2–3 months (Fig. 4A,B, shown at a higher scale in Fig.4C for clarity). *Lig4^Y288C^* mice showed a clear decrease in DSB levels from E17.5 to 2–3 months (Fig. 4B, blue columns). We did not detect any differences between 2-, 3- or 4-month-old mice suggesting that a steady state level is reached by 2 months (data not shown). At 2–3 months, DSB numbers in *Lig4^Y288C^* mice were approximately one tenth of the level at E14.5 (Fig. 4A,B). Perhaps surprisingly, the level of DSBs in *Atm^−^*^/−^ mice remained similar to the embryonic level, giving numbers slightly elevated compared to WT mice at P5, P15 and 2–3 months (Fig. 4A,B, light grey columns). Furthermore, the level of DSBs in the embryonic neocortex compared to in the isocortex in *Atm^−^*^/*−*^/*Lig4^Y288C^* mice also did not significantly decrease (Fig. 4A,B, red columns). Hence, DSB levels were similar at E14.5 and 2–3 months (approximately one 53BP1 focus per cell). Assuming that the induction of DSBs does not differ in these mice, these results suggest that ATM exerts an influence on the reparability of DSBs generated during neurogenesis.

In summary, these findings reveal a gradual decline in DSB levels in WT and *Lig4^Y288C^* mice from a high level during embryogenesis, when high DSB formation occurs, to a lower steady state level in adult mice. This decrease, particularly in the *Lig4^Y288C^* mice, is consistent with the notion that DSBs generated *in vivo* in the neocortex can be transmitted to newborn neuronal tissue and undergo slow repair.

### DSBs in the SVZ are lower than in the isocortex

Temporal analysis of DSBs in the SVZ revealed a pattern subtly distinct to that observed in the isocortex (Fig. 4B, compare SVZ and isocortex). For *Lig4^Y288C^* mice, DSB levels were statistically significantly lower in the SVZ than in the isocortex. This difference was particularly marked in the *Atm^−^*^/*−*^/*Lig4^Y288C^* mice, where numbers were lower than in the hippocampus or cerebellum (compare Fig. 4B and Fig. 3A). (Note that for *Atm^−^*^/*−*^/*Lig4^Y288C^* mice, the analysis was carried out at P20 and not P15 and is based on a single mouse; P5 mice were not examined.) This trend was not apparent in *Atm*^−/−^ mice. Taken together, these findings suggest that DSBs that arise in *Lig4^Y288C^* embryos undergo slow repair but that neuronal cells with DSBs can progress into the newborn SVZ and isocortex. The fact that the embryonic neocortical cells have approximately one 53BP1 focus per cell but can give rise to viable mice, substantiates the notion that cells with DSBs can be transmitted to newborn mice. Moreover, DSBs generated in *Atm^−^*^/*−*^/*Lig4^Y288C^* embryos either persist to adulthood or the neuronal tissue of such mice incurs further DSB formation. Most importantly, however, the results reveal that cells in the SVZ harbour fewer DSBs, raising the possibility that a process exists to diminish DSB levels in the SVZ, most noticeably at P5 and P15.

To assess whether this could be attributed to differences in the rate of repair between the two tissues, we assessed the rate of repair of DSBs induced by 100 mGy ionising radiation in the isocortex and SVZ of WT and *Lig4^Y288C^* mice, a dose which induces a similar number of DSBs to that observed at E14.5 in *Lig4^Y288C^* embryos (Fig. 2B; supplementary material Fig. S1A,B). For ethical considerations, the mice were only maintained for 15 h post ionising radiation. In WT and *Lig4^Y288C^* mice, we observed a similar rate of DSB repair in the isocortex, the SVZ and the cerebellum, although the repair was much delayed in *Lig4^Y288C^* mice compared to WT mice (Fig. 2B; supplementary material Fig. S1A). Thus, we conclude that the difference in DSB levels in the SVZ compared to the isocortex cannot be attributed to differences in the rate of repair of DSBs generated during neurogenesis.

To allow a comparison of the DSB repair kinetics with cultured cells and to assess the ATM dependency, we examined repair following 500 mGy ionising radiation in confluency-arrested primary mouse embryonic fibroblasts (MEFs) derived from WT and *Lig4^Y288C^* mice, and in *Lig4^Y288C^* MEFs treated with the ATM inhibitor (ATMi) KU55933 (Fig. 4D). Control experiments verified the linearity of the *in vitro* dose response following exposure to 50–500 mGy X-rays, and that 53BP1 and γH2AX foci numbers were identical (supplementary material Fig. S1C,D). 500 mGy induced ∼15 53BP1 foci per cell (approximately tenfold greater than endogenously generated DSBs during embryogenesis) (Fig. 4D). These DSBs were almost completely repaired by 12 days in *Lig4^Y288C^* MEFs (Fig. 4D, blue columns). *Atm^−^*^/*−*^ MEFs showed elevated unrepaired DSBs at 6 h post ionising radiation, and a persistent fraction of unrepaired DSBs could be detected at 12 days post ionising radiation, which is consistent with previous findings (Fig. 4D; supplementary material Fig. S1C, light grey columns) (Riballo et al., 2001). These have been shown to represent DSBs at heterochromatin regions, which have an essential requirement for ATM for repair (Goodarzi et al., 2008). Strikingly, the rate of DSB repair in ATMi-treated *Lig4^Y288C^* cells was slower than in non-ATMi treated cells but, nonetheless, only a small subset of DSBs remained by 12 days post ionising radiation (Fig. 4D; supplementary material Fig. S1C). These findings revealed a similar rate of repair of radiation-induced DSBs in cultured cells and *in vivo* (supplementary material Fig. S1A). However, the rate of loss of 53BP1 foci arising endogenously during embryogenesis in *Lig4^Y288C^* mice appeared slower than the repair of radiation-induced DSBs (compare Fig. 4A,B with supplementary material Fig. S1A). It is possible that *in vivo* cell division in the presence of DSBs impedes the rate of repair or that additional DSBs arise *in vivo*.

### Apoptosis is sensitively activated in the adult SVZ but not in other neuronal tissues

**Fig. 5.**
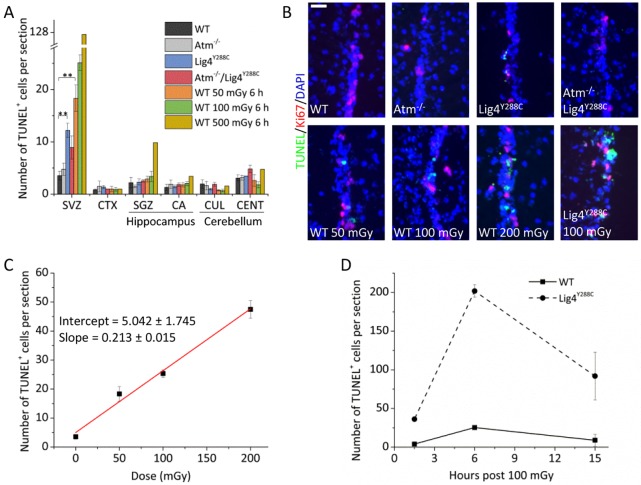
**Endogenous and ionising radiation-induced apoptosis is sensitively activated in the adult SVZ.** (A) TUNEL^+^ cells per section in untreated WT, *Atm^−^*^/*−*^, *Lig4^Y288C^*, *Atm^−^*^/*−*^/*Lig4^Y288C^* adult mice, and following irradiation with 50, 100 or 500 mGy X-rays. The region scored in each section encompassed the entire area of the tissue under analysis. CTX, Isocortex ; CA, Ammon's Horn; CUL, culmen; CENT, central lobule. (B) Representative images of a portion of the ventral SVZ stained for Ki67 (red), TUNEL (green) and DAPI (blue). Scale bar: 25 µm. The upper panel shows untreated mice and the lower panel figures were from mice killed at 6 h following irradiation with the indicated doses. (C) Dose–response and linear fitting of radiation-induced apoptosis in WT adult SVZ. (D) Timecourse of radiation-induced apoptosis in WT and *Lig4^Y288C^* adult SVZ exposed to 100 mGy X-rays. Data represent mean±s.e.m. (WT 50 mGy 6 h, *n*=3; WT 100 mGy 6 h, *n*=5; WT 500 mGy 6 h, *n*=1; WT 100 mGy 15 h, *n*=2; *Lig4^Y288C^* 100 mGy 1.5 h, *n*=2; *Lig4^Y288C^* 100 mGy 6 h, *n*=2; *Lig4^Y288C^* 100 mGy 15 h, *n*=2). **P≤0.01 (unpaired Student's *t*-test).

Adult neural stem cells have been shown to activate apoptosis after a high dose of ionising radiation but the sensitivity of activation has not been examined (Lazarini et al., 2009). To assess this, we quantified the endogenous apoptotic level in *Lig4^Y288C^* mice and the levels following exposure to a low dose of X-rays in WT mice, by measuring the total number of TUNEL^+^ cells in each brain region within the section. Using this approach, we were able to provide a correlation between apoptosis and DSB levels. No significant apoptosis was observed following ionising radiation exposure in WT mice or endogenously in the mutant strains in the adult hippocampus, cerebellum or isocortex (Fig. 5A, black columns). In marked contrast, substantial apoptosis was observed in the adult SVZ (Fig. 5A,B). Exposure of WT mice to ionising radiation gave a linear dose response with statistically significant apoptosis detectable after 50 mGy (Fig. 5C). Elevated endogenous apoptosis was also observed in the SVZ of *Lig4^Y288C^* mice at a level approximately half of that induced by 50 mGy in WT mice (Fig. 5A, compare blue and orange columns). This is consistent with the estimated DSB levels, which were similar to that induced by ∼20 mGy X-rays. Strikingly, no increase in apoptosis was observed in *Atm*^−/−^ mice despite DSB levels being similar to *Lig4^Y288C^* mice (Fig. 5A, compare blue and light grey columns). Furthermore, although DSBs were high in *Atm^−^*^/*−*^/*Lig4^Y288C^* mice, the level of apoptosis was lower than that observed in *Lig4^Y288C^* mice (Fig. 5A, compare blue and red columns). Apoptosis was also detected in the SGZ after exposure to 500 mGy, but not following lower doses; nor was it detected in the SGZ of *Lig4^Y288C^* mice. A timecourse analysis suggested that 6 h post exposure to ionising radiation was optimal for quantifying apoptosis in WT and *Lig4^Y288C^* mice (Fig. 5D).

Collectively, these findings demonstrate that apoptosis is sensitively activated in the adult SVZ, less sensitively in the SGZ and that there is no substantial activation in other neuronal tissues. Endogenous and radiation-induced apoptosis in the SVZ is largely ATM-dependent (Fig. 5A; supplementary material Fig. S2A).

### Developmentally regulated, ATM-independent apoptosis in the newborn SVZ

**Fig. 6.**
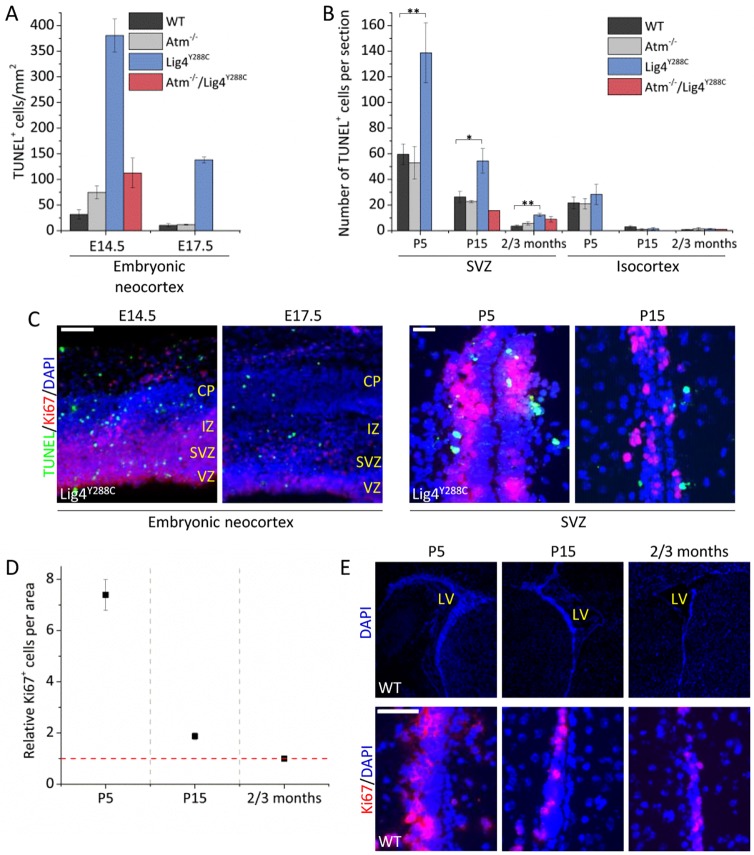
**Developmentally regulated ATM-independent apoptosis coupled with ATM-dependent DSB-induced apoptosis.** (A) TUNEL^+^ cells per mm^2^ in the embryonal neocortex at E14.5 and E17.5. (B) TUNEL^+^ cells per section in the postnatal (P5 and P15) and adult (2–3 months) SVZ and isocortex. *Atm^−^*^/*−*^/*Lig4^Y288C^* marked P15 represents P20. To allow direct comparison, both the number of TUNEL^+^ cells per section and per mm^2^ area were scored in the SVZ region demonstrating a similar profile (supplementary material Fig. S2B). (C) Representative images of the embryonal neocortex and postnatal SVZ of untreated *Lig4^Y288C^* mice stained for Ki67 (red), TUNEL (green) and DAPI (blue). Scale bars: 100 µm (left), 25 µm (right). (D) Quantification of Ki67^+^ cells per unit area (0.07 mm^2^) in the SVZ of P5, P15 and 2–3-month-old WT mice relative to the number of Ki67^+^ cells in the same area in 2–3-month-old WT mice. (E) Representative images of the lateral ventricle (LV) (top) of P5, P15 and 2–3-month-old WT mice stained for DAPI (blue) and a portion of the SVZ region (bottom) stained for Ki67 (red) and DAPI (blue). CP, cortical plate; IZ, intermediate zone; VZ, ventricular zone. Data represent mean±s.e.m. (*n* as described in Fig. 4). *P≤0.05; **P≤0.01 (unpaired Student's *t*-test).

We next undertook a temporal analysis of apoptosis in parallel to the DSB analysis by quantifying TUNEL^+^ cells in the embryonic neocortex and post birth in the SVZ and isocortex (Fig. 6A,B). Consistent with previous findings, we observed a low level of endogenous apoptosis in the WT neocortex at E14.5 and substantially higher apoptosis in *Lig4^Y288C^* embryos, demonstrating the marked sensitivity of the embryonic neocortical VZ/SVZ to DSB-induced apoptosis (Fig. 6A, black and blue columns). Interestingly, although *Atm*^−/−^ mice showed slightly higher apoptosis than WT mice, the levels were not further elevated in *Atm^−^*^/*−*^/*Lig4^Y288C^* mice despite high levels of DSBs, consistent with previous conclusions that apoptosis in the embryonic VZ/SVZ is predominantly ATM dependent (Fig. 6A, red columns) (Gatz et al., 2011). At E17.5, apoptosis had significantly decreased, as observed previously (Gatz et al., 2011), consistent with the reduction in DSB levels.

At P5, a low level of apoptosis was detected in the isocortex, which appeared similar in WT, *Atm*^−/−^ and *Lig4^Y288C^* mice; by P15 and adulthood there was no detectable apoptosis (Fig. 6B). In contrast, we observed substantial levels of apoptosis in the SVZ in all mice at P5 and P15, which was greater than observed at E17.5. The level was similar in WT and *Atm^−^*^/*−*^ mice and elevated in *Lig4^Y288C^* (Fig. 6B). To allow direct comparison, both the number of TUNEL^+^ cells per section and per mm^2^ area were scored in the SVZ region, and they showed a similar profile (supplementary material Fig. S2B). This pattern persisted at P15 although the overall level of apoptosis was reduced. By 2–3 months, the level of apoptosis was much lower, although it continued to be elevated in *Lig4^Y288C^* mice (Fig. 6B,C). The number of Ki67^+^ cells in the SVZ diminished from P5 to P15 to 2–3 months, suggesting that the ATM-independent process of apoptosis correlates with proliferation levels (Fig. 6D,E).

These findings provide the first evidence for ATM-independent apoptosis in the SVZ shortly after birth, and show that it is unrelated to the presence of DSBs because WT mice had low levels of DSBs at P5 compared to E17.5 yet a nearly tenfold increase in the level of apoptosis. Superimposed on this process, analysis of the *Lig4^Y288C^* mice also revealed the sensitive activation of DSB-induced ATM-dependent apoptosis in the SVZ. The slightly increased apoptosis at P5 in the isocortex could reflect either a similar process or the radial movement of some cells from the SVZ to the isocortex at this juvenile developmental stage.

## DISCUSSION

### Sensitive activation of apoptosis in the adult neural stem cells

**Fig. 7.**
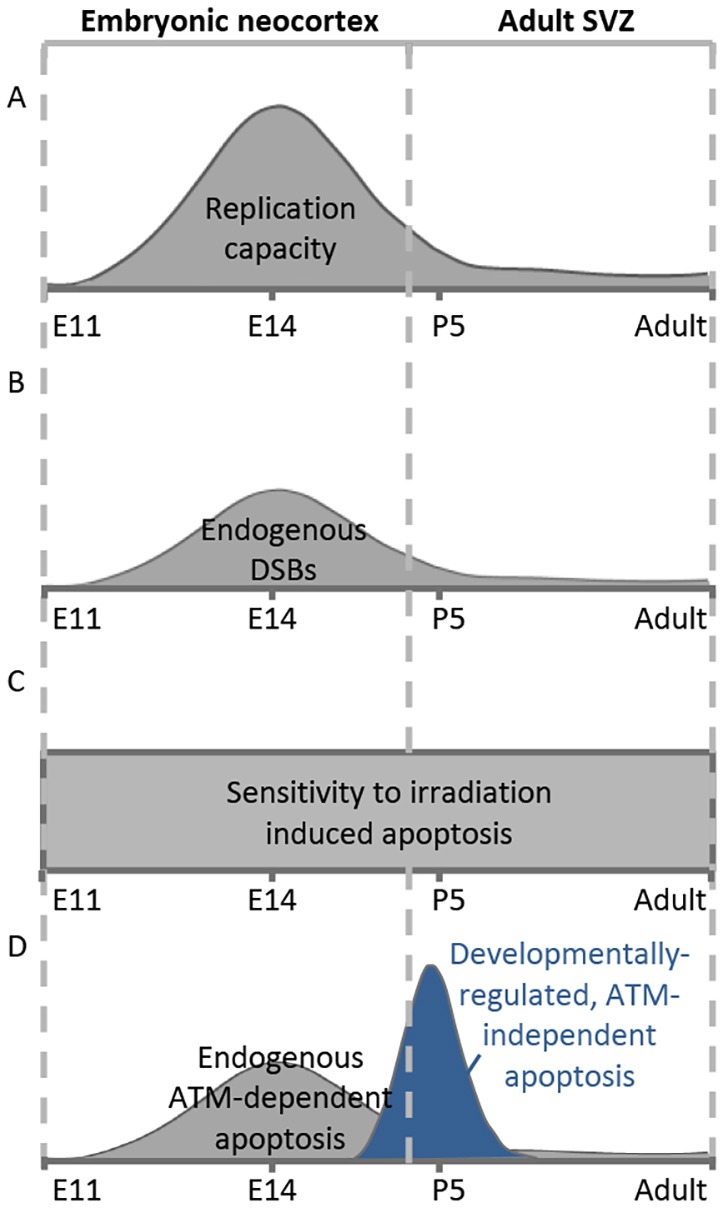
**Endogenous and ionising-radiation-induced DNA damage and apoptosis during embryonic and adult neurogenesis.** (A) In WT mice, the embryonic neocortex is characterised by high replication between E11 and E16.5, whereas the adult SVZ replicates slowly. (B) Replication-associated DNA DSBs arise during embryonic neurogenesis, reach a peak at ∼E14 and diminish temporally thereafter. The adult SVZ does not incur high endogenous DNA damage. (C) In terms of ionising-radiation-induced apoptosis, there is a similar, high sensitivity to DSB-induced apoptosis in the adult SVZ compared to the embryonic neocortex. (D) The elevated level of DSBs during embryonic neurogenesis triggers high endogenous apoptosis in a predominantly ATM-dependent manner. We also demonstrated the presence of a developmentally regulated stage of high apoptosis in the SVZ immediately post birth, which occurs independently of ATM, most likely reflecting the establishment of the adult stem cell compartment. There is little endogenous ATM-dependent apoptosis in the adult SVZ due to low DSB formation.

The embryonic neocortex is characterised by high proliferation from E11 to E16.5, high DSB damage and by sensitivity to DSB-induced apoptosis (Bayer et al., 1991; Gatz et al., 2011; Pontious et al., 2008; Saha et al., 2014). Here, we find that cells in the adult SVZ do not incur high levels of DSBs but sensitively activate apoptosis (Fig. 7). The comparison of DSB formation and apoptosis of the embryonic neocortex and adult SVZ in WT and *Lig4^Y288C^* mice is summarised in supplementary material Table S1. Apoptosis was activated in a linear dose-dependent manner in the SVZ of WT mice with 50 mGy causing detectable apoptosis (Fig. 5C). Endogenous DSBs and apoptosis in the SVZ in *Lig4^Y288C^* mice was similar to that detected following exposure to 15–20 mGy X-rays, suggesting that apoptosis can be activated by approximately one DSB per cell (assuming an induction of ∼25 DSBs per Gy). Taking DSB levels into account, we conclude that there is a similar or even enhanced sensitivity to DSB-induced apoptosis in the adult SVZ compared to the embryonic neocortical VZ/SVZ, demonstrating that this is an intrinsic feature of these compartments rather than an indirect consequence of rapid replication. Endogenous apoptosis was not detected in the SGZ of *Lig4^Y288C^* mice although exposure of WT mice to 500 mGy enhanced apoptosis (Fig. 5A). The marked sensitivity to DSB-induced apoptosis in the adult SVZ is important given the increased usage of computerized tomography (CT) scanning, when doses of 1–10 mGy can be received (Brenner and Hall, 2007). The functional role of the SVZ in the adult human brain remains unclear, but it has been proposed to promote tissue regeneration and cognitive plasticity, especially during infancy (Yang et al., 2011). Thus, the sensitivity to activate apoptosis could be important in situations when radiological procedures are used to assess neuronal damage and for scattered doses following cranial radiotherapy.

### The impact of ATM deficiency on DSB levels in the adult brain

The temporal analysis of DSBs and apoptosis in the mutant strains from E14.5 to 2–3 months post birth proved to be revealing. We compared DSBs and apoptosis in the embryonic neocortex with that in the postnatal isocortex and SVZ, tissues derived from the embryonic VZ/SVZ. *Lig4^Y288C^* and *Atm^−^*^/*−*^/*Lig4^Y288C^* mice harboured high DSB levels in both tissues at P5 and P15. Although it is difficult to eliminate the possibility that DSB generation continues at a high level post birth and gradually diminishes, these findings are consistent with the notion that cells with DSBs generated *in utero* can progress into the newborn mouse, where the DSBs can undergo slow repair. In *Lig4^Y288C^* mice, the rate of repair appears to be slower than observed in non-dividing cultured cells. This could arise if cells *in vivo* undergo cell division after DSB formation, which likely impacts upon their rate of repair. Alternatively, new DSBs could arise *in vivo.* Despite ongoing repair, by 2–3 months *Lig4^Y288C^* mice still have enhanced DSBs in all the neuronal tissues. A steady state level seems to have been reached by 2–4 months of age. Currently, we are unable to distinguish whether these represent residual unrepaired DSBs that arose in the embryo or whether DSBs also arise in the adult brain. Further work is required to address the level of DSB formation in the embryonic precursor compartments for the cerebellum and hippocampus. *Atm^−^*^/*−*^ mice also displayed enhanced DSBs in some neuronal tissues. *Atm^−^*^/*−*^ mice accrue some unrepaired DSBs by E14.5 although less than *Lig4^Y288C^* mice. The level of DSBs at E14.5 and in all post-birth stages appeared similar. A similar, but more striking and unexpected impact, was evident in the *Atm^−^*^/*−*^/*Lig4^Y288C^* double mutant mice. Thus, although the DSBs generated *in utero* in *Lig4^Y288C^* embryos undergo slow repair post birth, this does not appear to happen in an ATM-deficient background. It is unlikely that the role of ATM in regulating apoptosis can fully account for this because many cells in *Lig4^Y288C^* mice can escape apoptosis, but DSBs still undergo slow repair. Another possibility is that because ATM also regulates checkpoint arrest, the passage of damaged cells through the cell cycle checkpoints with unrepaired DSBs renders them irreparable. Although we cannot eliminate the possibility that *Atm^−^*^/*−*^ and *Atm^−^*^/*−*^/*Lig4^Y288C^* mice incur enhanced DSB formation during adulthood, the remarkable similarity in foci numbers in embryos and adult mice argues against this. Thus our findings raise the possibility that loss of ATM impedes the repair of DSBs generated during embryogenesis. Interestingly, despite enhanced embryonic lethality, the live *Atm^−^*^/*−*^/*Lig4^Y288C^* mice, although small, do not show marked neurodegeneration even though they harbour 53BP1 foci.

### The developing SVZ compartment undergoes high endogenous apoptosis

A new feature that we observed was a high level of apoptosis in the SVZ region at P5–P15. In WT mice, this process was ATM-independent, but the enhanced apoptosis at P15 in *Lig4^Y288C^* mice compared to in the double mutant mice suggests that an ATM-dependent process might also function (Fig. 7). The enhanced apoptosis is not simply due to the initiation of apoptosis in the embryo because the level is lower at E17.5 compared to P5. The SVZ region undergoes replication but diminishes in size during this period, suggesting the presence of a developmental process promoting cell loss; however, the precise mechanism has not been described (Sanai et al., 2011). Thus, we provide evidence that this stage of cell loss can proceed by apoptosis.

Interestingly, the *Lig4^Y288C^* neonates had fewer DSBs in the SVZ at P5 and P15 than observed in the corresponding isocortex. A similar and more evident relationship was seen in the single P15 and adult double mutant *Atm^−^*^/*−*^/*Lig4^Y288C^* mice. For *Atm^−^*^/*−*^/*Lig4^Y288C^* mice, the number of DSBs in the SVZ at 2–3 months was lower than in any other neuronal tissue. Although this impact cannot be entirely attributable to apoptosis, we speculate that a competitive environment, due to cell division coupled with high ATM-dependent and -independent apoptosis, in this developing stem cell compartment functions to diminish DNA breakage in the stem cells, thereby enhancing selection for the fittest stem cells. The process is not perfect, however, because cells with DSBs remain in the stem cell compartment. The more marked impact in the *Atm^−^*^/*−*^/*Lig4^Y288C^* mice might be partly attributable to the lack of repair in this genetic background. In contrast, DSBs in the *Lig4^Y288C^* mice undergo slow repair, which counteracts the impact of the selective process, which in this strain, is most apparent at P5 and P15. Interestingly, ATM-dependent apoptosis appeared to function in parallel to an ATM-independent process. Given that proliferation is ongoing, as shown by the high Ki67 levels, it is likely that the ATM-independent process represents ATR-dependent apoptosis, which functions following replication. Collectively, based on these findings, we speculate that apoptosis and/or a competitive environment in the developing stem cell compartment and surrounding niche functions to reduce the number of damaged stem cells, providing a mechanism to select for the fittest stem cells.

In summary, we demonstrate that the adult neural SVZ is exquisitely sensitive to DSB-induced apoptosis, with a sensitivity similar to that observed in the embryonic SVZ. The adult SVZ, however, is less vigorously replicating and is less prone to endogenous DSB formation demonstrating that sensitivity to DSB-induced apoptosis and rapid replication can be uncoupled. Finally, we identify a further stage of high sensitivity to apoptosis post birth during the development of the SVZ, and provide evidence to suggest that this process can serve to diminish the level of DNA breakage by removing cells with DSBs that arose during embryogenesis.

## MATERIALS AND METHODS

### Mice

*Lig4^Y288C^* (C57BL/6 background) and *Atm*^−/−^ (mixed 129/SV×C57BL/6 background) mice were as described previously (Barlow et al., 1996; Nijnik et al., 2007). *Atm^−^*^/*−*^ mice were crossed with *Lig4^Y288C^* (*Atm^−^*^/*−*^/*Lig4^Y288C^*) and developed on a mixed 129/SV×C57BL/6 background. *Atm^−^*^/*−*^/*Lig4^Y288C^* mice were born at sub-Mendelian frequencies based on the number of living mice born (expected *n*=60; observed *n*=4).

For embryonic analysis, the date of appearance of a vaginal plug was considered as embryonic day 0.5 (E0.5) and the day of birth as postnatal day 0 (P0). Embryos were taken at E14.5 and E17.5. Mice were designated adult from P50 and examined at 2–4 months post birth. Controls (WT) were wild-type littermates of either sex. All animal experiments were carried out in accordance with accepted standards of animal welfare approved by the United Kingdom Home Office and complied with the Animals (Scientific Procedures) Act 1986.

### Primary MEF isolation

Primary MEFs were prepared from E13.5 embryonic mouse tissues and cultured as described previously (Nijnik et al., 2007).

### Mouse irradiation

The whole body of the adult mice were exposed to X-rays using an AGO HS X-Ray System at 250 kV potential and 500 mGy/min dose rate.

### Cell irradiation and drug treatment

Confluence-arrested primary MEFs were irradiated on a glass slide using a ^137^Cs γ-ray source. When indicated, cells were incubated with the ATM inhibitor KU55933 (KuDos Pharmaceuticals, London, UK) at a final concentration of 10 µM for 30 min before ionising radiation. During repair, cells were re-exposed to 10 µM KU55933 for 30 min every 48 h.

### Immunofluorescence staining

Preparation and immunostaining of tissue sections were performed as described (Gatz et al., 2011). The primary antibodies used were against: 53BP1 (rabbit, 1:500; cat. no. A300-272A; Bethyl, Montgomery, TX), γH2AX (Ser139, mouse, 1:500; cat. no. 05-636; Merck Millipore, Billerica, MA), Ki67 (rat, 1:500; cat. no. 14-5698-82; eBioscience, San Diego, CA), lamin B (goat, 1:500; cat. no. sc-6216; Santa Cruz Biotechnology, Dallas, TX) and cleaved caspase-3 (rabbit, 1:200; cat. no. 9579S; Cell Signaling, Danvers, MA). Appropriate secondary antibodies were used and were conjugated to FITC (donkey anti-rabbit-IgG, 1:200; cat. no. 711-095-152; Jackson ImmunoResearch, West Grove, PA), Alexa Fluor 488 (donkey anti-rabbit-IgG, 1:500; cat. no. A-21206; Life Technologies, Carlsbad, CA), Alexa Fluor 594 (donkey anti-rat-IgG,1:500; cat. no. A-21209; Life Technologies) and DyLight 594 (donkey anti-rabbit-IgG; cat. no. A120-208D4; Bethyl).

For fluorescent staining of MEFs, cells were fixed for 10 min with 3% PFA and 2% sucrose in PBS and permeabilized for 2.5 min with 0.2% Triton X-100 in PBS. Primary antibodies were diluted in 2% BSA in PBS for 45 min at room temperature. Cells were then rinsed in PBS and incubated for 45 min at room temperature in the dark with secondary antibodies conjugated to FITC (goat anti-mouse-IgG, 1:200; cat. no. F0257; Sigma, St Louis, MO) or Cy3 (sheep anti-rabbit-IgG, 1:200; cat. no. C2306; Sigma).

### TUNEL assay

Terminal deoxynucleotidyltransferase-mediated dUTP nick-end labeling (TUNEL) staining was performed as described by the manufacturer (Roche, Basel, Switzerland). After primary and secondary antibody staining, the TUNEL reaction mixture was added and samples were incubated for 1 h at 37°C in a darkened humidified chamber. Slides were counterstained with DAPI and mounted with Vectashield.

### Image analysis and quantification

The counts of 53BP1 foci per cell were scored in the IZ and CP region of the embryonic neocortex at E14.5 and E17.5. Quantification was performed as described previously (Saha et al., 2014). At least two sections were quantified per embryo with a minimum of 100 cells scored for each section. TUNEL quantification was carried out as TUNEL^+^ cells per mm^2^ in a defined area of the developing neocortex at E14.5 and E17.5 (VZ, SVZ, IZ and CP). Mutant or WT embryo forebrains were less than twofold different in size.

For counts of 53BP1 foci per cell in the postnatal and adult mouse tissues, 100 cells per region per section from at least two sections per mouse were scored. TUNEL quantification involved scrolling the entire area of each brain region to quantify TUNEL^+^ cells per section. To quantify proliferation in the SVZ of P5, P15 and 2–3-month-old mice, all Ki67^+^ cells in a 0.07 mm^2^ area close to the ventral SVZ were scored and the ratio was related to the number of Ki67^+^ cells in the same area in 2–3-month-old WT mice. Images were acquired with a Zeiss Axioplan or a Nikon eclipse/E400 microscope and analysed using Simple PCI software and ImageJ.

### Statistical analysis

At least two mice of each genotype and treatment were quantified (averaged from at least two sections per mouse). No mice were excluded. Bar and symbol plots represent mean values of independent replicates, and error bars represent s.e.m. Statistical analysis was carried out using a two-tailed unpaired Student's *t*-test or a one-way or two-way ANOVA. Linear and exponential models were used for data fitting (Origin 8.6).
